# Impaired recognition of interactive intentions in adults with autism spectrum disorder not attributable to differences in visual attention or coordination via eye contact and joint attention

**DOI:** 10.1038/s41598-024-58696-2

**Published:** 2024-04-09

**Authors:** Mathis Jording, Arne Hartz, David H. V. Vogel, Martin Schulte-Rüther, Kai Vogeley

**Affiliations:** 1 Cognitive Neuroscience, Institute of Neuroscience and Medicine (INM-3), Forschungszentrum Jülich, Jülich, Germany; 2 Department of Psychiatry, Faculty of Medicine, University Hospital Cologne, University of Cologne, Cologne, Germany; 3 Child Neuropsychology Section, Department of Child and Adolescent Psychiatry, Psychosomatics, and Psychotherapy, University Hospital RWTH, Aachen, Germany; 4 Department of Neurology, Faculty of Medicine, University Hospital Cologne, University of Cologne, Cologne, Germany; 5 Department of Child and Adolescent Psychiatry, Center for Psychosocial Medicine - University Hospital Heidelberg, Ruprechts-Karls University Heidelberg, Heidelberg, Germany; 6 Department of Child and Adolescent Psychiatry and Psychotherapy, University Medical Center Göttingen, Georg-August University Göttingen, Göttingen, Germany

**Keywords:** Human behaviour, Social behaviour

## Abstract

Altered nonverbal communication patterns especially with regard to gaze interactions are commonly reported for persons with autism spectrum disorder (ASD). In this study we investigate and differentiate for the first time the interplay of attention allocation, the establishment of shared focus (eye contact and joint attention) and the recognition of intentions in gaze interactions in adults with ASD compared to control persons. Participants interacted via gaze with a virtual character (VC), who they believed was controlled by another person. Participants were instructed to ascertain whether their partner was trying to interact with them. In fact, the VC was fully algorithm-controlled and showed either interactive or non-interactive gaze behavior. Participants with ASD were specifically impaired in ascertaining whether their partner was trying to interact with them or not as compared to participants without ASD whereas neither the allocation of attention nor the ability to establish a shared focus were affected. Thus, perception and production of gaze cues seem preserved while the evaluation of gaze cues appeared to be impaired. An additional exploratory analysis suggests that especially the interpretation of contingencies between the interactants’ actions are altered in ASD and should be investigated more closely.

## Introduction

One of the core symptoms of Autism Spectrum Disorder (ASD) are impairments in communication and social interactions^1,2^. Especially nonverbal communication abilities and specifically the perception, production, and interpretation of gaze cues are in the focus of different screening and diagnostic procedures for ASD^3,4^ and have received much attention in research^5–14^. Alterations in processing and responding to gaze cues are already relevant for the diagnosis in childhood^15^, they persist throughout adolescence^16–18^ into adulthood^6,19–21^. However, as set out below, results for adults with ASD are still unexpectedly sparse and inconclusive as to their ability to communicate via gaze i.e. to reach a common ground in a gaze interaction.

Traditionally and following a naïve clinical intuition, individuals with ASD have been reported to focus less on socially relevant stimuli or, in case of looking at human faces, focus more on the mouth region than the eyes^22–24^. The background of this phenomenon has been discussed extensively^14^. According to the amygdala theory, a hypoactive amygdala in persons with ASD might fail to flag the eyes as socially relevant which thus do not attract visual attention^25^. However, a review of neuroscientific data^26^ seems to be more in favor of the competing eye-avoidance theory, according to which amygdala hyperactivity causes unpleasant levels of arousals which leads persons with ASD to avoid eye-contact^27^.

Furthermore, the reduction in the focus on the eye region might also only manifest for situations with a certain social richness or interactional affordance. Several studies using static or non-interactive stimuli were not able to replicate earlier findings^7,9, 28, 29^ while group differences were most prominent during ongoing dynamic, socially enriched encounters, e.g. eyes directed at the viewer vs. averted eyes^21^, dynamic depictions of faces^30^ or faces of persons in interaction with others^11,31^.

Aside from mere visual attention and eye contact, a more complex aspect of gaze interactions is the ability to follow the partners gaze and to engage in joint attention. Typically, gaze following skills are ontogenetically closely linked to the development of mentalizing abilities^32^. In children with ASD, reflexive gaze following as the response to joint attention (RJA) initiated by a partner seems to be preserved^33,34^. However, the initiation of joint attention (IJA), where a person tries to direct the gaze of the counterpart towards an object, has been reported to be impaired^35–39^. It seems that some of these deficits persist into adolescence^16–18^. However, until today, only very few studies have investigated gaze following or joint attention in adults with ASD. One study that systematically investigated gaze following did not find any impairments in ASD regarding oculomotor control, attention orienting or executive function in a non-social condition^20^. In the social condition adults with ASD successfully initiated JA were less accurate in responding to JA, which – as the authors suggest – could be based on difficulties in monitoring the partners intentions and recognizing and correctly interpreting communicative gaze behavior like eye contact that preceded the JA bids. Congruently, observing initial eye contact between two faces increased the tendency to follow with their gaze in control participants but not adults with ASD^19^. Other studies did not find any behavioral group specific differences in RJA but changed activation patterns in areas associated with socio-cognitive processing in adults^40,41^ and adolescents^17^ with ASD. With regard to the IJA condition, It was speculated that especially the requirement of self-initiation or self-motivation might be difficult to reproduce in experimental setups^41^. This might explain the missing group differences in the IJA condition in adults with ASD^20,41^. A more detailed summary and discussion of this issue can be found in Mundy (2018)^42^.

It is not sufficient for the development of shared intentions that two persons look at an object simultaneously^43^ or plan joint actions^44^. It is crucial that both have to be mutually aware of the attentional focus of the other. This is sometimes reflected in the distinction between ‘joint attention’, where only one of the interactants has to be aware of the partners focus and ‘shared attention’, where both interactants are mutually aware^45,46^. Mutual awareness might be ensured by ascertaining that eye movements of the counterpart are actual responses to one´s own behavior, e.g. based on spatial^47^ and/or temporal associations^48^. Potentially, this process also requires multiple repetitions, i.e. only when the partner repeatedly follows with their gaze within a certain time window the impression of mutual awareness emerges^46,49^. Whether persons with ASD are impaired in their ability to recognize such social contingencies is a question that gained some attention recently^50–54^. In extremely minimalistic interactions in one-dimensional environments, adults with ASD did not show impairments in their ability to detect contingencies between their own and their partners behavior^53^. However, in gaze interactions adults with ASD seemed to be less likely to recognize that a counterpart was following their gaze^50^. Correspondingly, Northrup^52^ hypothesizes that infants and small children with ASD do initially detect contingencies between their behavior and their environment. However, especially in the social realm the complexity of interactions and contingencies between interactants would increase fast which would overextend the children’s capacities, depriving the children from learning opportunities.

This study investigates to which degree visual attention and the ability to coordinate with a counterpart via eye contact and joint attention diverge in ASD and whether these impairments could predict potential difficulties in recognizing interactive intentions. Participants with and without ASD were instructed to judge in repeated trials, whether a partner, displayed as a virtual character (VC), was trying to interact with them or not. The VC was controlled by the agent-platform “TriPy”^55^. TriPy processes eye-tracking data and controls an anthropomorphic VC in real-time to create a gaze-contingent interaction partner. It incorporates five possible states of attention in triadic interactions (constituted by two interactants and one or more objects in a shared environment) identified in a recent review^56^. The five states are: ‘partner-oriented’ (attention directed towards the partner); ‘object-oriented’ (attention directed towards objects); ‘introspective’ (attention directed towards inner (bodily) experiences and disengaged from the outside world); ‘responding joint attention’ (active following of the partner’s gaze); ‘initiating joint attention’ (proactive attempts to lead the partner’s gaze towards objects of one’s own choice).

The first three states are non-interactive, i.e. the behavior is completely independent of the partner, these states are implemented in TriPy based purely on predefined probabilities. The latter two states are interactive where the behavior of one partner is contingent upon the behavior of the other, i.e. the agent follows the participants gaze (RJA) or looks at the participant, waiting for them to establish eye contact before looking at an object and again waiting for the participant to follow (IJA). Participants were not aware of the algorithm controlling their partners behavior but were told the cover story that the VC would represent another participant (in fact a confederate who did not have any influence on the behavior of the VC which was fully controlled by an algorithm). The VC assumed one pseudo-randomly chosen gaze state^56^ in each trial, with participants not being instructed about the current gaze state, the behavioral repertoire of the VC or the differences between gaze states.

Our approach is in accordance with a number of recent advances in interactive study designs investigating behavior during face-to-face communication instead of focusing on the isolated observation or production of gaze cues between two detached partners^21,57–60^. However, instead of having two participants directly interact with each other^58,60^ or letting a participant interact with an experimenter^21,57, 59^, we used an VC based system. The benefit of a VC as interaction partner is that it provides full experimental control over the different degrees of responsiveness in every trial.

We analyzed the participants’ allocation of visual attention by measuring for how long they looked at different areas of interest (AoI): the VCs eyes (referred to as “eyes”), the VCs face excluding the eye region (referred to as “face”), and the objects in the environment (referred to as “objects”). Our hypothesis (I) was that participants with ASD compared to control participants would spent less time on the VC’s eyes in relation to the other AoIs.

Second, we assessed the ability to coordinate with an interaction partner via gaze by identifying and counting all situations in which the two interactants (participant and VC) fixated the same target. Instances of shared focus encompassed eye contact (participant and VC both looking at each other’s eyes) or joint attention (both looking at the same object). Note, that shared focus instances were identified purely on a behavioral basis and thus do not necessarily imply (mutual) awareness of each other’s focus (in contrast to the term “shared attention” mentioned above). We hypothesized (II) that in persons with ASD, less instances of shared focus would be established. We did not have specific hypothesis as to whether eye contact or joint attention would be more closely associated with the intention to interact, thus the summary as shared focus instances. However, in order to potentially elucidate effects, we also briefly report on an exploratory differential analysis of eye contact and joint attention instances.

Lastly, we analyzed the participants decisions of whether the VC was trying to interact with them or not. Our hypothesis (III) was that persons with ASD would be impaired in their ability to recognize that the partner was trying to interact (either by following the participants gaze or by initiating JA themselves) with them. Correspondingly, we expected smaller differences of interactive ratings between the non-interactive and the interactive VC in the ASD group compared to the control group. In an additional exploratory analysis, we aimed at elucidating whether differences in the recognition of social contingencies in form of shared focus events could potentially explain group differences in the recognition of interactive intentions. As it is unclear, whether and to which degree differences between RJA and IJA impairments reported for children with ASD persist into adulthood, we did not have specific hypothesis regarding group specific differences in interactivity ratings between these states. However, as an exploratory analysis we analyzed the relationship between the establishment of shared focus events and interactivity ratings separately for all five agent states.

## Methods

All methods and procedures summarized below are described in full detail in Jording et al.^61^.

### Participants

26 subjects with ASD, diagnosed in the Autism Outpatient Clinic at the Department of Psychiatry, University Hospital Cologne, were recruited via the Outpatient Clinic. The diagnostic procedure started by a screening with the Autism-Spectrum-Quotient^62^ and was only continued when patients exceeded the cut-off value (> 32). The diagnosis then had to be confirmed in two independent and extensive clinical interviews by two separate professional clinicians according to ICD-10 criteria^2^. After the exclusion of 5 participants (due to missing data or mistrust of the cover story) the remaining 21 subjects (6 identifying as female, 15 as male; aged 22–54, mean = 40.86, SD = 10.36) were compared to a group of 24 control subjects (with an overlap to the population reported in^61^), without any record of psychiatric or neurological illnesses (10 identifying as female, 14 as male; aged 23–58, mean = 39.00, SD = 12.76). Demographic data and the Autism-Spectrum-Quotient (AQ; Baron-Cohen et al., 2001b) were obtained from all subjects. None of the participants from the control group exceeded the commonly preferred cut-off of > 32, while all participants from the ASD group did, indicating a clear difference in the expression of autistic symptoms between both groups. Both groups of participants had comparable educational backgrounds in terms of years of education which also served as a proxy for intellectual capacities (ASD: mean = 17.77, SD = 5.93; Controls: mean = 16.27, SD = 4.28). Informed consent was obtained from all subjects. Subjects received a monetary compensation (10€ per hour). This study was presented to and approved by the ethics committee of the Medical Faculty of the University of Cologne and strictly adhered to the declaration of Helsinki. All experiments were conducted in accordance with relevant guidelines and regulations.

### Procedure and tasks

**Figure 1:**
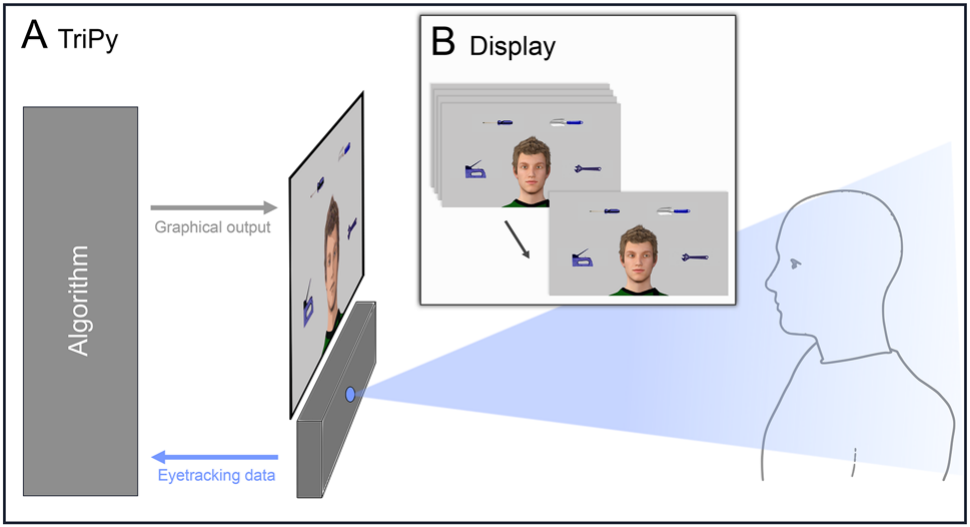
Illustration of the technical setup and the participants’ perspective during the experiment (adapted from Jording et al., 2019). (**A**) Illustration of a participant interacting with the agent controlled by the platform TriPy. (**B**) The behavior of the agent created by TriPy as seen from the perspective of the participant.

To make the subjects believe that they were participating in an ongoing social encounter, they were introduced to a confederate of the same sex in a briefing room prior to the start of the experiment. Participants were informed that they would interact with their interaction partner via a computer, with their partner being seated in a different room. After this introduction, participants were separated from the confederate and brought to the testing room, where they received detailed written and oral experimental instructions. Participants were informed that both partners were to be represented on their computer screen by an identical generic male VC serving as an avatar of the partner and that during the interaction they would see their partner’s avatar instead of her/his real face. Furthermore, they would only be allowed to communicate with their partner via gaze, while all other communication channels (e.g. speech, gestures, facial expressions) would not be transmitted or displayed. Importantly, the avatar displayed on the participants’ screen was always and entirely being controlled by the computer algorithm (Fig. 1A^61^;). In addition to the avatar, four trial-wise changing objects were displayed on the screen at fixed locations in the avatar´s field of view (Fig. 1B).

In full accordance with Jording et al.^61^, participants had to take two alternative roles, the Observation-Role (ObR) and the Action-Role (AcR), in alternating blocks. In total, 6 blocks were presented, 3 ObR blocks containing 16 ObR trials per block and 3 AcR blocks containing 10 AcR trials per block. Trials within blocks were presented in randomized order and separated by short breaks of 2–6 s. Blocks were separated by breaks of approx. 3 min allowing a short phase of rest for the participant and re-calibration of the eye-tracker.

In the ObR condition participants had to ascertain by button-press whether their partner was trying to interact with them or not. The ObR was the primary target condition of our analysis with the focus on the participants’ responses (“interactive” or “not interactive”) and the respective preceding gaze behavior. Trials lasted until participants’ response but maximally 30 s.

During AcR, for each trial one out of five states (equating to the five social gaze states^56,61^) was pseudo-randomly chosen, and participants were asked to 1: concentrate on their partner, 2: concentrate on the objects; 3: concentrate on their breathing; 4: to let the partners gaze guide them; or 5: to guide the partner with their gaze. Each trial lasted 30 s. The sole purpose of the AcR condition was to make participants believe the cover story by suggesting a balanced study design with the same tasks for both participants. Data of AcR were not analysed.

The experiment was followed by a post-experimental questionnaire asking participants on 6-point scales about the difficulty of the two tasks, as well as the naturalness of the interaction and the quality of the technical realization of the VC’s eye movements. Additional open text items asked for the participants’ experience during the experiment as well as their assumptions about the purpose of the study. An additional interview by the experimenter inquired whether participants believed the cover story. Answers to the written open text questions and during the post-experimental interview were carefully screened for indications of mistrust in the cover story (e.g. indicating a lack of conviction of having interacted with a real person). While none of the written answers indicated any such suspicions, in the interview two participants indicated that they had at least questioned the announcement to have interacted with a real human. Both participants were excluded from further analysis (as mentioned earlier).

### Setup, agent-platform and pilot study

The VC’s behavior and graphical output was controlled by the newly developed agent-platform ‘TriPy’^55^ already applied before^61^. TriPy adapts the behavior of a VC to the behavior of the participant in real-time (‘gaze-contingent’^56,63^). While earlier studies relied on pre-determined behavior^64–68^ in TriPy the VCs’ behavior in the non-interactive states is implemented on a probabilistic basis. For the purpose of this study, we implemented five social gaze states (with behavioral parameters being empirically informed by a pilot study^61^). In the non-interactive states, the VC was not responsive to the participant (although occasionally looking at the participant which could result in incidental eye contact). In the interactive states, the VC either “responded” to the participant by consequently following their gaze or tried or “initiate” joint attention by repeatedly initiating eye contact before then shifting towards one of the objects and briefly waiting for the participant to follow (For video examples of all states see supplementary of^61^).

When participants had to ascertain the interactivity of the VC (ObR mode), the VC states were balanced with 24 trials (50%) of interactive and 24 trials (50%) of non-interactive states. In total, each participant encountered each non-interactive state (PO, OO and INT) 8 times and each interactive state (RJA + IJA) 12 times in the ObR mode, pseudo-randomly distributed over 3 blocks. In the AcR mode, where participants were instructed to engage in a specific gaze state, each of the five states appeared 6 times in total (2 per block in random order). Whenever the participants were in interactive-states, the VC reacted with the complementary behavior (RJA with IJA; IJA with RJA) and during non-interactive states with another non-interactive state with all combinations of VC and participant states appearing equally often. The eye-tracker ran at a sampling rate of 120 Hz and an accuracy of 0.5° (Tobii TX300; Tobii Technology, Stockholm, Sweden). A 23’’ monitor (screen resolution: 1920*1080 pixels) mounted on top of the eye-tracker was used as the display (Fig. 1A). Participants were seated at a distance ranging between 50 and 70 cm from the monitor and gave their responses (during ObR) via a keyboard with the marked buttons “J” (for “yes”) and “N” (for “no”).

### Data preprocessing and statistical analysis

From 2160 trials total in the ObR condition (45 participants with 48 trials each), 92 trials were excluded due to missing responses or response times exceeding 30 s, another 432 trials were excluded because of more than 20% of missing gaze data. After trial exclusion, 1636 valid trials remained for statistical analysis. Response, eye-tracking data and questionnaire data were pre-processed and statistically analyzed using the software R (version 3.6.2^69^). Analysis followed the procedure described previously in Jording et al.^61^. Response time data were logarithmized to reduce skewness and more closely resemble a normal distribution. Eye-tracking and response data were modelled as (generalized) linear mixed effects models (“lme4” package^70^) with random intercepts for participants. The effects of individual factors were analyzed in likelihood ratio tests of differently saturated models, by testing whether adding a factor to a model significantly increased the models’ fit to the data. Factors were added to the model and tested in the order of mentioning. Where the analysis revealed significant interaction effects, individual factor level combinations were compared in Tukey post-hoc tests correcting for multiple comparisons (‘multcomp’ package^71^).

To assess the allocation of attention, we computed ‘relative fixation durations’ for the AoIs ‘eyes’, ‘face’ (excluding the eyes), and ‘objects’ (all four objects) as the cumulative fixation duration on an AoI in relation to the overall trial duration. We analyzed differences between the AoIs (factor “*aoi*”), the diagnostic groups (“*group*”) and the interaction aoi*group.The establishment of shared focus instances was analyzed as the sum of eye contact and joint attention instances in a poisson regression (generalized mixed effects model with log link function). Eye contact was defined as situations in which participant and the VC both looked at each other’s eyes, joint attention as situations in which both looked at the same object. We assessed the effect of interactive VC states vs. non-interactive states (factor “*interactivity*”), the diagnostic groups (factor “*group*”), and the interaction interactivity*group. The participants’ ability to recognize interactive intentions was analyzed in form of their interactivity ratings at the end of each trial. Data were modeled in logistic regression models (generalized mixed effects model with logit link function). We assessed the effects *interactivity*, *group*, and interactivity*group (an analysis of the effects of individual states can be found in supplementary material S3.1.4–S3.16).

## Results

### Distribution of visual attention

**Figure 2:**
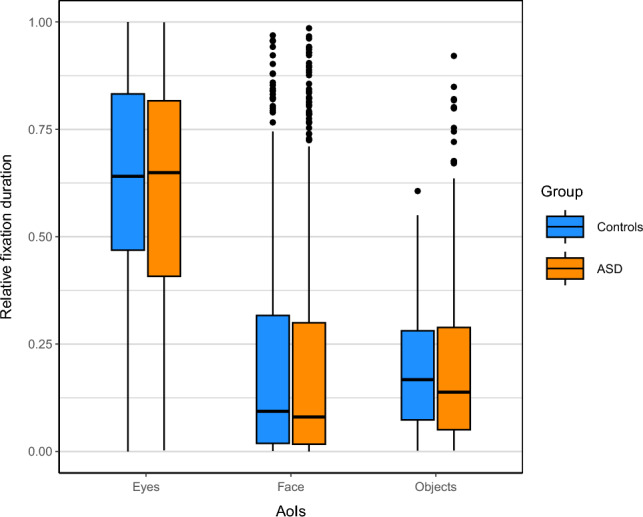
Boxplot of the distribution of the participant’s visual attention measured as relative fixation durations, i.e. the portion of time spent on the different AoIs (eyes, face, objects) per trial for both diagnostic groups (blue: control participants / orange: ASD participants). Diagnostic groups do not differ significantly in their distribution of visual attention.

In order to assess group differences in visual attention, we analyzed gaze behavior during ObR (Fig. 2), specifically with regard to the distribution of the visual attention between the AoIs (Eyes, Face, Object). Model fits for relative durations (as the portion of the total time that was spent on the specific AoI, ranging from 0 to 1) were significantly improved by including the factor *aoi* (X^2^ (2) = 2144.33, *p* < 0.001). The factor *group* did not significantly improve the model fit, neither directly (X^2^(1) = 1.39, *p* < 0.239), nor as part of the interaction aoi*group (X^2^(2) = 5.83, *p* = 0.054; supplementary tables S1.1 & S1.2). Thus, the results do not support hypothesis I of deficits in attention allocation in ASD patients.

### Establishment of shared focus (eye contact and joint attention)

**Figure 3:**
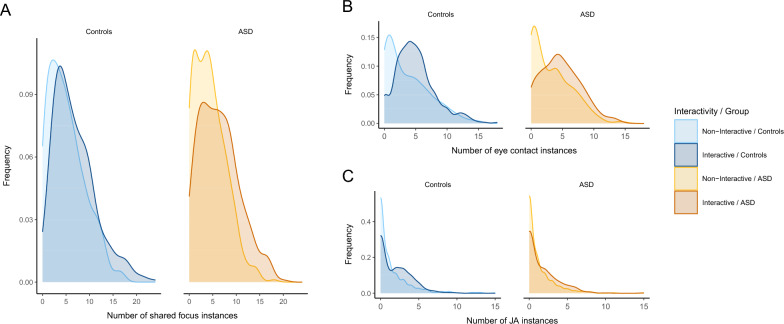
Frequency of shared foci combined (eye contact + joint attention; (**A**)) and separate for eye contact (**B**) and joint attention (**C**) between participant and agent per trial for control participants (left, blue) and ASD participants (right, orange) and non-interactive agent (light colors) vs. interactive agent (dark colors). Diagnostic groups do not differ with regard to non-interactive states or response times.

Next, we analyzed whether the frequency of the establishment of shared focus (eye contact or joint attention) differed between groups and interactive vs. non-interactive VCs (Fig. 3A). The fit for the prediction of the number of shared focus instances was significantly improved by *interactivity* (X^2^(1) = 230.91, *p* < 0.001), but not by *group* (X^2^(1) = 1.68, *p* = 0.195) or the interaction interactivity*group (X^2^(1) = 2.68, *p* = 0.101; supplementary table S2.1.1). When the VC was interactive compared to non-interactive, the predicted incidence rate of shared foci was increased by a factor of 1.33 (CL = 1.26–1.41) for control subjects and 1.42 (1.33 * 1.07 (CL = 0.99–1.16)) for ASD subjects (supplementary table S2.1.2). Again, results do not support hypothesis II of reduced capabilities to establish instances of shared focus in interactions.

A separate exploratory analysis of eye contact (Fig. 3B) and joint attention (Fig. 3C) instances revealed the same pattern with the amount of eye contact instances significantly increasing with *interactivity* (X^2^(1) = 166.23, *p* < 0.001), but not *group* (X^2^(1) = 1.05, *p* = 0.306) or interactivity*group (X^2^(1) = 2.71, *p* = 0.100; supplementary tables S2.2.1 & S2.2.2) and the amount of JA instances significantly increasing with *interactivity* (X^2^(1) = 64.71, *p* < 0.001), but not *group* (X^2^(1) = 1.02, *p* = 0.313) or interactivity*group (X^2^(1) = 0.21, *p* = 0.650; supplementary tables S2.3.1 & S2.3.2).

### Recognizing interactive intentions

**Figure 4:**
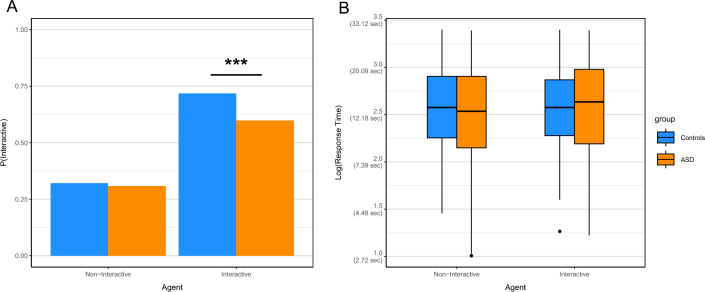
Plots of (**A**) mean interactivity ratings and (**B**) mean logarithmic response times (sec values in parentheses) for diagnostic groups (blue: control persons / orange: ASD participants) and non-interactive (left) vs. interactive (right) agent states. A: Asterisks indicate significant post-hoc comparisons (* < .05, ** < .01, *** < .001), dashed line indicates the 50% guessing rate). ASD participants have significantly smaller detection rates for interactive states compared to controls participants. Diagnostic groups do not differ with regard to non-interactive states or response times.

We analyzed differences in participants’ experiences of interactivity with respect to VC states between both diagnostic groups (Fig. 4A). Likelihood ratio tests of logistic regression models revealed significant effects of the VCs *interactivity* (X^2^(1) = 211.41, *p* < 0.001) and a significant interaction effect for interactivity*group (X^2^(1) = 5.17, *p* = 0.023), but no significant main effect of *group* (X^2^(1) = 2.67, *p* = 0.103; supplementary table S3.1.1). The predicted odds ratio for identifying the partners interactive state as interactive was decreased for the ASD subjects compared to the control participants by a factor of 0.61 (Cl = 0.39–0.93, supplementary table S3.1.2). In a Tukey post-hoc test participants with ASD and control participants differed significantly in recognizing interactive (M = 0.57, SE = 0.22, z = − 2.56, *p* = 0.048) but not in recognizing non-interactive VC states (supplementary table S3.1.3). For an exploratory analysis of group specific effects of individual agent states see supplementary tables S3.1.4 & S.3.1.5 and supplementary table S3.1.6 for post-hoc pairwise comparisons.

For the logarithmic response times (Fig. 4B), likelihood ratio tests did not reveal any significant effects of *interactivity* (X^2^(1) = 1.84, *p* = 0.175), *group* (X^2^(1) = 0.22, *p* = 0.640), or interactivity*group (X^2^(1) = 1.48, *p* = 0.115; supplementary table S3.2).

The inclusion of *gender* of participants as a factor did not significantly improve model fits for the mean interactivity ratings (ASD: F(1, 19) = 0.82, *p* = 0.377; controls: F(1, 22) = 2.41, *p* = 0.135; supplementary tables S3.3.1 & S3.3.2) or mean response times (ASD: F(1, 19) = 0.37, *p* = 0.553; controls: F(1, 22) = 0.47, *p* = 0.501; supplementary tables S3.3.3 & S3.3.4) in either group.

Results of diminished interactivity ratings for interactive VCs in the ASD group support hypothesis iii) of impaired abilities in ASD to recognize the partner’s intention to establish an interaction. Response times give no reason to believe that this effect might due to the fact that persons with ASD merely require more time for their assessment.

### Exploratory analysis of the role of contingencies between interactants

**Figure 5:**
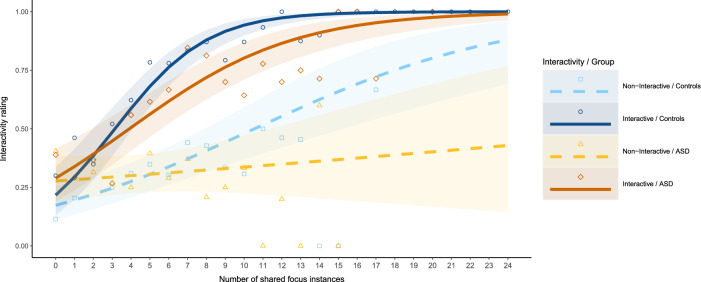
Interactivity ratings for differing numbers of shared foci instances (eye contact or joint attention) between participant and agent per trial, separately for control participants (blue) and ASD participants (orange) and a non-interactive agent (light colors) vs. an interactive agent (dark colors). Mean rates (triangles, diamonds, squares, and circles) and model predictions (lines) with 95% confidence intervals (ribbons) of interactivity ratings. The establishment of shared foci on average predicts the participants’ interactivity ratings. This effect is especially strong for interactive compared to non-interactive agents and for control participants compared to ASD participants.

In an exploratory analysis, we investigated, to what extent the number of shared focus instances could predict the interactivity ratings depending on whether the VC was actually trying to interact or not (Fig. 5). We started from the model with best fit for the prediction of the participants’ response, including the predictors *interactivity*, *group* and the interaction interactivity*group (see section ‘Recognizing interactive intentions’). We tested, whether additionally including the number of *shared focus* instances would, by themselves or via interaction effects, improve the model fit. The prediction of the participants response was significantly improved when including *shared focus* (X^2^(1) = 118.72, *p* < 0.001), the interaction interactivity*shared focus (X^2^(1) = 39.47, *p* < 0.001) and the interaction group*shared focus (X^2^(1) = 12.95, *p* < 0.001). The interaction interactivity*group*shared focus instances did not significantly improve the fit (X^2^(1) = 0.69, *p* = 0.407; supplementary table S4.1.1). In control subjects each shared focus increased the odds ratio of interactive ratings by 1.17 (CL = 1.10–1.24) when the VC was non-interactive. When the VC was interactive, the odds ratio increased with each shared focus by 1.47 (1.17 * 1.26 (CL = 1.17–1.35)). In ASD subjects, the odds ratio increase per shared focus instance was lower by the factor 0.87 (CL = 0.80–0.94)) compared to control subjects (supplementary table S4.1.2).

A separate analysis of the effects of eye contact (without joint attention) revealed a comparable picture, i.e. significant model fit improvements by the factor eye contact (X^2^(1) = 41.18, *p* < 0.001), interactivity*eye contact (X^2^(1) = 26.50, *p* < 0.001) and group*eye contact (X^2^(1) = 5.30, *p* = 0.021) but not interactivity*group*eye contact (X^2^(1) = 0.01, *p* = 0.914; supplementary table S4.2.1 & S4.2.2). Interestingly, for the effect of joint attention, in addition to the effects of joint attention (X^2^(1) = 101.45, *p* < 0.001), interactivity*joint attention (X^2^(1) = 76.55, *p* < 0.001) and group*joint attention (X^2^(1) = 7.91, *p* = 0.005) also the three-way interaction interactivity*group*joint attention (X^2^(1) = 8.92, *p* = 0.003) significantly improved the model fit (supplementary table S4.3.1 & S4.3.2). This would suggest that the effect that joint attention instances increase the odds of interactive ratings especially strongly, if the agent was indeed interactive, was diminished in persons with ASD by a factor of 0.61 (CL = 0.43–0.85).

## Discussion

The present study investigated the ability of adults with ASD to interact with a partner in an extended, naturalistic gaze interaction. It assessed the ability of adults with ASD to recognize interactive intentions of their partner in gaze interactions. Furthermore, it allowed for the analysis of visual attention and the coordination between the interactants through the establishment of eye contact and joint attention as events of shared focus. This was achieved by making use of new platform for extended and unrestricted human-VC gaze interactions^55,61^.

The participants’ visual attention was operationalized as the proportional duration with which different AoIs were fixated. The analysis did not reveal significant differences between the two groups. Thus, we have no reasons to assume a general or pervasive impairment in the allocation of visual attention to socially relevant information in gaze interactions in adults with ASD. This is contrary to results about diminished attention to early socially relevant stimuli and especially human eyes in ASD^22–24^. Considering that in the past interactive or socially enriched settings were found to more clearly distinguish visual attention in ASD^7,9, 28, 29^, we had expected a pronounced difference. However, the task explicitly draws the attention of participants to the eye region by instructing to guide the other or to be guided by them via gaze. Previous studies have demonstrated that such explicit attentional mechanisms can attenuate behavioral differences for facial processing^72,73^ and gaze processing^17^ in ASD.

We further investigated the coordination between the two interactants in form of the occurrence of shared focus instances (eye contact, joint attention). Both diagnostic groups did not differ significantly with regard to the frequency of shared focus events. In both groups, more shared focus events occurred in interactive as compared to non-interactive trials without any discernible influence of the diagnostic group. Thus, these results do not support the hypothesis of a diminished ability to engage in eye contact or joint attention in adults with ASD. A detailed analysis of the literature reveals a less clear picture regarding gaze processing and joint attention in ASD than commonly expected. Gaze direction processing, i.e. the ability to estimate the gaze angle and line of sight of another person, has been found to be impaired in ASD^74–76^. However, in one study, children with ASD were able to trace the line of sight of another person – be it by less conventional strategies^77^. Furthermore, no differences were found in the ability to detect changes in gaze direction between adolescent and adult ASD and control participants^78^. Previous results on gaze cueing in ASD, i.e. the effect that onlookers automatically shift their (covert) attention in accordance with the gaze direction of observed eyes, were heterogeneous as well^79,80^.

Although both attention allocation and the establishment of shared focus (eye contact and joint attention) are preserved, the subsequent processing step of the interpretation of the arising social contingencies are impaired in persons with ASD. This corresponds to similar results in the domains person perception^81^ and animacy experience^82^, bringing forth deficits in the evaluation of social stimuli but not in the mere detection. It is also in line with fMRI studies in which control participants and participants with ASD did not necessarily react differently to observing joint attention but still showed different activation patterns in areas related to social-cognitive processing^41,83^. Similarly, altered activation patterns in areas of the “social brain” despite comparable behavioral performance were observed in ASD adolescents, suggesting less elaborated processing of gaze cues in social contexts^17^. A promising follow-up to further test changes in processing and interpretation of gaze cues in ASD with a more focused and higher powered study was suggested by an anonymous reviewer. Assuming no general impairment in establishing joint attention in ASD, the frequency of joint attention instances should not differ between groups in interactions with object-oriented agents. However, in interactions with an IJA agent where the agent additionally uses eye-contact to signal its communicative intention, persons with ASD would not profit as much from this signal and subsequently would not show the same increase in joint attention instances as expected for healthy participants.

The exploratory analysis of the relationship between shared focus instances and interactivity ratings yielded some interesting insights into the differential evaluation of gaze by ASD participants as compared to control participants. It seems that the probability of an ‘interactive’ rating generally increases with the number of shared focus events. This would suggest that eye contact and joint attention were interpreted as a signal for an interactive situation. Furthermore, it would corroborate earlier findings demonstrating that shared attention, i.e. mutual awareness of the joint effort to coordinate attention, is established by alternating between eye contact and joint attention^46^.

Interestingly, the effect of eye contact and joint attention on the interactivity ratings was stronger when the VC was interactive, i.e. was reacting to the participant in a contingent fashion. Thus, it seems that participants’ impression of the VCs interactive intentions was influenced by the contingencies between them and the VC. This begs the question, whether participants were also aware of these contingencies. In previous studies, healthy participants^65,84^ and participants with ASD^50^ were able to detect and react to a VC following their gaze without becoming aware of the dependencies. With regard to differences between diagnostic groups, we found that the frequency of eye contact and especially joint attention predicted interactivity ratings more reliably for control participants than for ASD participants. This is in concordance with the generally reduced sensitivity to gaze cues reported for ASD^75,81, 85^. However, other studies did not find impairments in the detection of social contingencies in ASD^53^. Future studies should focus on the question whether the detection or the evaluation of social contingencies could be responsible for the reduced impression of interactive intentions in ASD.

So far, the majority of studies on social gaze behavior in ASD focused on isolated aspects of gaze behavior and examined these under highly controlled experimental conditions. These studies have provided already a very detailed picture of some of the elements and building blocks of gaze interaction in ASD and constitute a foundation for further advances in the field. However, the knowledge acquired from these reductionist approaches is fragmentary and very specific for a particular experimental setup, whereas the embedding in a dynamic and more complex context is missing. In this study, we followed a new, holistic approach in which we observed the unfolding encounter while participants engaged in gaze-based interaction. This allowed us to systematically differentiate behaviors related to different parts of the task and compare them between groups.

## Limitations

It is important to take into account some limitations of the design (see also^61^). We deliberately focused on gaze interaction and restricted the interaction to this channel. More available communication channels might allow for faster and more accurate evaluations of the interaction. With regards to the differences between control and ASD subjects, it should be noted that both groups differed in gender distribution. We did not systematically manipulate VCs’ gender. However, we did not have any specific hypothesis for gender differences, and we did not find any significant effects for gender on mean interactivity ratings or mean response times for either of the two groups. We also did not systematically control for the IQ of participants and while we can rule out cases of intellectual disabilities in our sample, the possibility of an effect of IQ remains. It has to be emphasized that the design does not allow to test a causal relationship or rule out additional differences between groups that might affect the performance in recognizing intentions.

## Conclusion

ASD participants did not show any perceptual differences in visual attention or mere detection during gaze encounters as compared to control persons, nor did the emerging interactions differ in the establishment of eye contact or joint attention. Nonetheless, ASD participants evaluated the perceived cues differently when compared to control participants and recognized interactive intentions less frequently. This finding has implications for the investigation of interaction disturbances in ASD as well as for the development of diagnostic and therapeutic instruments. Instead of a simple passive observation and quantification of patients’ behaviors, a holistic and socially contextualized consideration of patients’ inner experience during interaction is mandatory. The newly developed human-agent interaction platform TriPy, with its implementation of different gaze states as a holistic taxonomy of triadic gaze interactions, has proven to be a reliable tool for this kind of investigation. Furthermore, it constitutes a promising basis for a future diagnostic or therapeutic instrument in clinical contexts.

### Supplementary Information

Supplementary Information.

## Data Availability

The ethics approval for this study does not allow for the publication of raw data. Requests to access the datasets should be directed to Mathis Jording, m.jording@fz-juelich.de.
