# Supplementary material for: Impaired recognition of interactive intentions in adults with autism spectrum disorder not attributable to differences in visual attention or coordination via eye contact and joint attention
Source: Sci Rep. 2024 Apr 9;14:8297. doi: 10.1038/s41598-024-58696-2 (PMC11004189; doi:10.1038/s41598-024-58696-2)
Supplement: Supplementary file 1 — Supplementary Information. [file 41598_2024_58696_MOESM1_ESM.docx]

1. **Distribution of visual attention**

Analysis of the effect of *aois*, the agents’ *interactivity* and diagnostic *group* on relative fixation durations

Table S1.1: Likelihood ratio tests

| Model | Df | logLik | deviance | Chisq | Sig. |
| --- | --- | --- | --- | --- | --- |
| Null | 3 | -973.40 | 1946.79 | - | - |
| AoI | 5 | 98.77 | -197.54 | 2144.33 | .000 |
| AoI + Group | 6 | 99.46 | -198.93 | 1.39 | .239 |
| AoI * Group | 8 | 102.38 | -204.76 | 5.83 | .054 |

Table S1.2: Parameters of the model “AoI * Group”

|  |  | | |  |
| --- | --- | --- | --- | --- |
| *Predictors* | *Estimates* | *CI* |  |  |
| (Intercept) | 0.62 | 0.59 – 0.64 |  | |
| AoI [Face] | -0.43 | -0.45 – -0.40 |  | |
| AoI [Object] | -0.44 | -0.46 – -0.41 |  | |
| Group [ASD] | -0.04 | -0.08 – -0.00 |  | |
| AoI [Face] * Group [ASD] | 0.02 | -0.01 – 0.06 |  | |
| AoI [Objects] * Group [ASD] | 0.04 | 0.01 – 0.08 |  | |
| **Random Effects** | | | |  |
| σ^2^ | 0.05 | | |  |
| τ_00_ _subj.ID_ | 0.00 | | |  |
| ICC | 0.05 | | |  |
| N _subj.ID_ | 45 | | |  |
| Observations | 3829 | | |  |
| Marginal R^2^ / Conditional R^2^ | 0.423 / 0.452 | | |  |

1. **Establishment of shared focus (eye contact and joint attention)**

**2.1 Shared focus (eye contact + joint attention)**

Analysis of the effect of the agents’ *interactivity* and diagnostic *group* on the number of *shared focus* (eye contact + joint attention) instances

Table S2.1.1: Likelihood ratio tests

| Model | Df | logLik | deviance | Chisq | Sig. |  |
| --- | --- | --- | --- | --- | --- | --- |
| Null | 1 | -4422.50 | 8845.00 | - | - |  |
| Interactivity | 3 | -4307.04 | 8614.08 | 230.91 | .000 |  |
| Interactivity + Group | 4 | -4306.20 | 8612.40 | 1.68 | .195 |  |
| Interactivity * Group | 5 | -4304.86 | 8609.72 | 2.68 | .101 |  |

Table S2.1.2: Parameters of the model “Interactivity * Group”

|  |  | | |
| --- | --- | --- | --- |
| *Predictors* | *Incidence Rate Ratios* | *CI* |  |
| (Intercept) | 4.61 | 3.72 – 5.70 |  |
| Agent State [Interactive] | 1.33 | 1.26 – 1.41 |  |
| Group [ASD] | 0.78 | 0.57 – 1.07 |  |
| Agent State [Interactive] * Group [ASD] | 1.07 | 0.99 – 1.16 |  |
| **Random Effects** | | | |
| σ^2^ | 0.19 | | |
| τ_00_ _subj.ID_ | 0.27 | | |
| ICC | 0.59 | | |
| N _subj.ID_ | 45 | | |
| Observations | 1636 | | |
| Marginal R^2^ / Conditional R^2^ | 0.075 / 0.622 | | |

**2.2 Eye contact**

Analysis of the effect of the agents’ *interactivity* and diagnostic *group* on the number of eye contact instances

Table S2.2.1: Likelihood ratio tests

| Model | Df | logLik | deviance | Chisq | Sig. |  |  |
| --- | --- | --- | --- | --- | --- | --- | --- |
| Null | 2 | -4137.62 | 8275.24 | - | - |  |  |
| Interactivity | 3 | -4054.51 | 8109.01 | 166.23 | .000 |  |  |
| Interactivity + Group | 4 | -4053.98 | 8107.97 | 1.05 | .306 |  |  |
| Interactivity * Group | 5 | -4052.63 | 8105.25 | 2.71 | .100 |  |  |

Table S2.2.2: Parameters of the model “Interactivity * Group”

|  |  | | |
| --- | --- | --- | --- |
| *Predictors* | *Incidence Rate Ratios* | *CI* |  |
| (Intercept) | 3.37 | 2.71 – 4.19 |  |
| Agent State [Interactive] | 1.32 | 1.24 – 1.41 |  |
| Group [ASD] | 0.81 | 0.59 – 1.12 |  |
| Agent State [Interactive] * Group [ASD] | 1.08 | 0.98 – 1.19 |  |
| **Random Effects** | | | |
| σ^2^ | 0.24 | | |
| τ_00_ _subj.ID_ | 0.28 | | |
| ICC | 0.53 | | |
| N _subj.ID_ | 45 | | |
| Observations | 1636 | | |
| Marginal R^2^ / Conditional R^2^ | 0.059 / 0.560 | | |

**2.3 Joint attention**

Analysis of the effect of the agents’ *interactivity* and diagnostic *group* on the number of JA instances

Table S2.3.1: Likelihood ratio tests

| Model | Df | logLik | deviance | Chisq | Sig. |  |  |
| --- | --- | --- | --- | --- | --- | --- | --- |
| Null | 2 | -2791.03 | 5582.06 | - | - |  |  |
| Interactivity | 3 | -2758.68 | 5517.36 | 64.71 | .000 |  |  |
| Interactivity + Group | 4 | -2758.17 | 5516.34 | 1.02 | .313 |  |  |
| Interactivity * Group | 5 | -2758.07 | 5516.13 | 0.21 | .650 |  |  |

Table S2.3.2: Parameters of the model “Interactivity * Group”

|  |  | | |
| --- | --- | --- | --- |
| *Predictors* | *Incidence Rate Ratios* | *CI* |  |
| (Intercept) | 4.61 | 3.72 – 5.70 |  |
| Agent State [Interactive] | 1.33 | 1.26 – 1.41 |  |
| Group [ASD] | 0.78 | 0.57 – 1.07 |  |
| Agent State [Interactive] * Group [ASD] | 1.07 | 0.99 – 1.16 |  |
| **Random Effects** | | | |
| σ^2^ | 0.19 | | |
| τ_00_ _subj.ID_ | 0.27 | | |
| ICC | 0.59 | | |
| N _subj.ID_ | 45 | | |
| Observations | 1636 | | |
| Marginal R^2^ / Conditional R^2^ | 0.075 / 0.622 | | |

1. **Recognizing interactive intentions**

**3.1 Interactivity ratings**

Analysis of the effect of the agents’ *interactivity* and diagnostic *group* on the participants probability to rate the agent interactive

Table S3.1.1: Likelihood ratio tests

| Model | Df | logLik | deviance | Chisq | Sig. |
| --- | --- | --- | --- | --- | --- |
| Null | 2 | -1116.18 | 2232.35 | - | - |
| State | 3 | -1010.47 | 2020.94 | 211.41 | .000 |
| State + Group | 4 | -1009.14 | 2018.28 | 2.67 | .103 |
| State * Group | 5 | -1006.55 | 2013.11 | 5.17 | .023 |

Table S3.1.2: Parameters of the model “Interactivity * Group”

|  |  | | |
| --- | --- | --- | --- |
| *Predictors* | *Odds Ratios* | *CI* |  |
| (Intercept) | 0.46 | 0.35 – 0.62 |  |
| Agent State [Interactive] | 5.89 | 4.36 – 7.95 |  |
| Group [ASD] | 0.94 | 0.61 – 1.44 |  |
| Agent State [Interactive] * Group [ASD] | 0.61 | 0.39 – 0.93 |  |
| **Random Effects** | | | |
| σ^2^ | 3.29 | | |
| τ_00_ _subj.ID_ | 0.26 | | |
| ICC | 0.07 | | |
| N _subj.ID_ | 45 | | |
| Observations | 1636 | | |
| Marginal R^2^ / Conditional R^2^ | 0.152 / 0.215 | | |

Table S3.1.3: Tukey corrected post-hoc test of the model “Interactivity * Group”

| Comparison | Estimate | SE | z | p |
| --- | --- | --- | --- | --- |
| Interactive.Controls - Non-Interactive.Controls | 1.77 | 0.15 | 11.58 | .000 |
| Non-Interactive.ASD - Non-Interactive.Controls | -0.07 | 0.22 | -0.30 | .990 |
| Interactive.ASD - Non-Interactive.Controls | 1.21 | 0.22 | 5.53 | .000 |
| Non-Interactive.ASD - Interactive.Controls | -1.84 | 0.22 | -8.24 | .000 |
| Interactive.ASD - Interactive.Controls | -0.57 | 0.22 | -2.56 | .048 |
| Interactive.ASD - Non-Interactive.ASD | 1.27 | 0.16 | 8.01 | .000 |

Analysis of the effect of the agents *state* and diagnostic *group* on the participants probability to rate the agent interactive

Table S3.1.4: Likelihood ratio tests

| Model | Df | logLik | deviance | Chisq | Sig. |
| --- | --- | --- | --- | --- | --- |
| Null | 2 | -1116.18 | 2232.35 | - | - |
| Agent State | 6 | -952.19 | 1904.38 | 327.98 | .000 |
| Agent State + Group | 7 | -950.78 | 1901.55 | 2.83 | .093 |
| Agent State * Group | 11 | -941.65 | 1883.30 | 18.25 | .001 |

Table S3.1.5: Parameters of the model “State * Group”

|  | **response** | | | |
| --- | --- | --- | --- | --- |
| *Predictors* | *Odds Ratios* | *CI* |  | |
| (Intercept) | 0.99 | 0.66 – 1.49 | |  |
| agent state [PO] | 0.39 | 0.23 – 0.64 | |  |
| agent state [OO] | 0.22 | 0.13 – 0.38 | |  |
| agent state [RJA] | 1.29 | 0.83 – 1.99 | |  |
| agent state [IJA] | 9.01 | 5.20 – 15.62 | |  |
| group [ASD] | 0.61 | 0.34 – 1.12 | |  |
| agent state [PO] × group [ASD] | 1.52 | 0.73 – 3.18 | |  |
| agent state [OO] × group [ASD] | 2.67 | 1.24 – 5.76 | |  |
| agent state [RJA] × group [ASD] | 1.18 | 0.62 – 2.24 | |  |
| agent state [IJA] × group [ASD] | 0.51 | 0.24 – 1.06 | |  |
| **Random Effects** | | | | |
| σ^2^ | 3.29 | | | |
| τ_00_ _subj.ID_ | 0.30 | | | |
| ICC | 0.08 | | | |
| N _subj.ID_ | 45 | | | |
| Observations | 1636 | | | |
| Marginal R^2^ / Conditional R^2^ | 0.257 / 0.319 | | | |

Table S3.1.6: Tukey corrected post-hoc test of the model “State * Group”

| Comparison | Estimate | SE | z | p |
| --- | --- | --- | --- | --- |
| PO.Controls - INT.Controls | -0.95 | 0.26 | -3.72 | .007 |
| OO.Controls - INT.Controls | -1.51 | 0.28 | -5.49 | .001 |
| RJA.Controls - INT.Controls | 0.25 | 0.22 | 1.13 | .979 |
| IJA.Controls - INT.Controls | 2.20 | 0.28 | 7.84 | .001 |
| INT.ASD - INT.Controls | -0.49 | 0.30 | -1.60 | .838 |
| PO.ASD - INT.Controls | -1.02 | 0.31 | -3.26 | .035 |
| OO.ASD - INT.Controls | -1.02 | 0.32 | -3.22 | .040 |
| RJA.ASD - INT.Controls | -0.07 | 0.28 | -0.25 | 1 |
| IJA.ASD - INT.Controls | 1.03 | 0.29 | 3.50 | .016 |
| OO.Controls - PO.Controls | -0.56 | 0.29 | -1.96 | .612 |
| RJA.Controls - PO.Controls | 1.20 | 0.24 | 5.10 | .001 |
| IJA.Controls - PO.Controls | 3.15 | 0.29 | 10.74 | .001 |
| INT.ASD - PO.Controls | 0.47 | 0.32 | 1.48 | .894 |
| PO.ASD - PO.Controls | -0.07 | 0.32 | -0.22 | 1 |
| OO.ASD - PO.Controls | -0.07 | 0.33 | -0.20 | 1 |
| RJA.ASD - PO.Controls | 0.88 | 0.30 | 2.98 | .079 |
| IJA.ASD - PO.Controls | 1.98 | 0.31 | 6.48 | .001 |
| RJA.Controls - OO.Controls | 1.76 | 0.26 | 6.88 | .001 |
| IJA.Controls - OO.Controls | 3.71 | 0.31 | 11.96 | .001 |
| INT.ASD - OO.Controls | 1.03 | 0.33 | 3.10 | .057 |
| PO.ASD - OO.Controls | 0.49 | 0.34 | 1.45 | .905 |
| OO.ASD - OO.Controls | 0.49 | 0.34 | 1.45 | .906 |
| RJA.ASD - OO.Controls | 1.44 | 0.31 | 4.62 | .001 |
| IJA.ASD - OO.Controls | 2.54 | 0.32 | 7.90 | .001 |
| IJA.Controls - RJA.Controls | 1.95 | 0.26 | 7.45 | .001 |
| INT.ASD - RJA.Controls | -0.74 | 0.29 | -2.56 | .224 |
| PO.ASD - RJA.Controls | -1.27 | 0.30 | -4.28 | .001 |
| OO.ASD - RJA.Controls | -1.27 | 0.30 | -4.22 | .001 |
| RJA.ASD - RJA.Controls | -0.32 | 0.27 | -1.21 | .968 |
| IJA.ASD - RJA.Controls | 0.78 | 0.28 | 2.81 | .126 |
| INT.ASD - IJA.Controls | -2.69 | 0.34 | -8.00 | .001 |
| PO.ASD - IJA.Controls | -3.22 | 0.34 | -9.36 | .001 |
| OO.ASD - IJA.Controls | -3.22 | 0.35 | -9.28 | .001 |
| RJA.ASD - IJA.Controls | -2.27 | 0.32 | -7.16 | .001 |
| IJA.ASD - IJA.Controls | -1.17 | 0.33 | -3.59 | .012 |
| PO.ASD - INT.ASD | -0.54 | 0.28 | -1.94 | .625 |
| OO.ASD - INT.ASD | -0.53 | 0.28 | -1.90 | .652 |
| RJA.ASD - INT.ASD | 0.42 | 0.24 | 1.72 | .774 |
| IJA.ASD - INT.ASD | 1.52 | 0.26 | 5.94 | .001 |
| OO.ASD - PO.ASD | 0.00 | 0.29 | 0.01 | 1 |
| RJA.ASD - PO.ASD | 0.95 | 0.25 | 3.76 | .006 |
| IJA.ASD - PO.ASD | 2.05 | 0.27 | 7.72 | .001 |
| RJA.ASD - OO.ASD | 0.95 | 0.26 | 3.69 | .008 |
| IJA.ASD - OO.ASD | 2.05 | 0.27 | 7.60 | .001 |
| IJA.ASD - RJA.ASD | 1.10 | 0.23 | 4.80 | .001 |

**3.2 Response times**

Analysis of the effect of the agents’ *interactivity* and diagnostic *group* on logarithmized response times

Table S3.2: Likelihood ratio tests

| Model | Df | logLik | deviance | Chisq | Sig. |
| --- | --- | --- | --- | --- | --- |
| Null | 3 | -730.38 | 1460.76 | - | - |
| Interactivity | 4 | -729.46 | 1458.92 | 1.84 | .175 |
| Interactivity + Group | 5 | -729.35 | 1458.70 | 0.22 | .640 |
| Interactivity * Group | 6 | -728.11 | 1456.22 | 1.48 | .115 |

- 1. **Effect of gender**

Analysis of the effect of the gender on the participants probability to rate the agent interactive (Table S3.5 & S3.6) and logarithmized response times (Table S3.7 & S3.8) for ASD group (Table S3.5 & S3.7) and control group (Table S3.6 & S3.8)

Table S3.3.1: Likelihood ratio tests (interactivity rating / ASD group)

| Model | Res.Df | RSS | Df | Sum of Squares | F | Pr(>F) |
| --- | --- | --- | --- | --- | --- | --- |
| Null | 20 | 0.47 | - | - | - | - |
| Gender | 19 | 0.45 | 1 | 0.02 | 0.82 | .377 |

Table S3.3.2: Likelihood ratio tests (interactivity rating / control group)

| Model | Res.Df | RSS | Df | Sum of Squares | F | Pr(>F) |
| --- | --- | --- | --- | --- | --- | --- |
| Null | 23 | 0.37 | - | - | - | - |
| Gender | 22 | 0.34 | 1 | 0.04 | 2.41 | .135 |

Table S3.3.3: Likelihood ratio tests (log(response times) / ASD group)

| Model | Res.Df | RSS | Df | Sum of Squares | F | Pr(>F) |
| --- | --- | --- | --- | --- | --- | --- |
| Null | 20 | 522.16 | - | - | - | - |
| Gender | 19 | 512.30 | 1 | 9.86 | 0.37 | .553 |

Table S3.3.4: Likelihood ratio tests (log(response times) / control group)

| Model | Res.Df | RSS | Df | Sum of Squares | F | Pr(>F) |
| --- | --- | --- | --- | --- | --- | --- |
| Null | 23 | 228.52 | - | - | - | - |
| Gender | 22 | 223.76 | 1 | 4.77 | 0.47 | .501 |

1. **Exploratory analysis of the role of contingencies between interactants**

**4.1 Effect of shared focus instances (eye contact + joint attention)**

Analysis of the effect of the agents’ *interactivity*, diagnostic *group* and the number of *shared focus* instances on the participants probability to rate the agent interactive

Table S4.1.1: Likelihood ratio tests

| Model | Df | logLik | deviance | Chisq | Sig. |
| --- | --- | --- | --- | --- | --- |
| Interactivity * Group | 5 | -1006.55 | 2013.11 | - | - |
| Interactivity * Group + Shared Focus | 6 | -947.20 | 1894.39 | 118.72 | .000 |
| Interactivity * Group + Interactivity * Shared Focus | 7 | -927.46 | 1854.92 | 39.47 | .000 |
| Interactivity * Group + Interactivity * Shared Focus + Group * Shared Focus | 8 | -920.99 | 1841.97 | 12.95 | .000 |
| Interactivity * Group * Shared Focus | 9 | -920.64 | 1841.29 | 0.69 | .407 |

Table S4.1.2: Parameters of the model
“Interactivity * Group + Interactivity * Shared Focus + Group * Shared Focus

|  |  | | |
| --- | --- | --- | --- |
| *Predictors* | *Odds Ratios* | *CI* | |
| (Intercept) | 0.20 | 0.12 – 0.32 | |
| Agent State [Interactive] | 1.55 | 0.97 – 2.50 | |
| Group [ASD] | 2.03 | 1.06 – 3.89 | |
| Shared Focus | 1.17 | 1.10 – 1.24 | |
| Agent State [Interactive] * Group [ASD] | 0.60 | 0.38 – 0.96 |  |
| Agent State [Interactive] * Shared Focus | 1.26 | 1.17 – 1.35 | |
| Group [ASD] * Shared Focus | 0.87 | 0.80 – 0.94 | |
| **Random Effects** | | | |
| σ^2^ | 3.29 | | |
| τ_00_ _subj.ID_ | 0.51 | | |
| ICC | 0.13 | | |
| N _subj.ID_ | 45 | | |
| Observations | 1636 | | |
| Marginal R^2^ / Conditional R^2^ | 0.352 / 0.439 | | |

**4.2 Effect of eye contact instances**

Analysis of the effect of the agents’ *interactivity*, diagnostic *group* and the number of eye contact instances on the participants probability to rate the agent interactive

Table S4.2.1: Likelihood ratio tests

| Model | Df | logLik | deviance | Chisq | Sig. |
| --- | --- | --- | --- | --- | --- |
| Interactivity * Group | 5 | -1006.55 | 2013.11 | - | - |
| Interactivity * Group + Eye Contact | 6 | -985.96 | 1971.92 | 41.18 | .000 |
| Interactivity * Group + Interactivity * Eye Contact | 7 | -972.71 | 1945.42 | 26.50 | .000 |
| Interactivity * Group + Interactivity * Eye Contact + Group * Eye Contact | 8 | -970.06 | 1940.12 | 5.30 | .021 |
| Interactivity * Group * Eye Contact | 9 | -970.05 | 1940.11 | 0.01 | .914 |

Table S4.2.2: Parameters of the model
“Interactivity * Group + Interactivity * Eye Contact + Group * Eye Contact

|  |  | |  |
| --- | --- | --- | --- |
| *Predictors* | *Odds Ratios* | *CI* |  |
| (Intercept) | 0.33 | 0.22 – 0.49 |  |
| Agent State [Interactive] | 2.29 | 1.48 – 3.53 |  |
| Group [ASD] | 1.35 | 0.77 – 2.36 |  |
| Eye Contact | 1.09 | 1.03 – 1.16 |  |
| Agent State [Interactive] * Group [ASD] | 0.64 | 0.41 – 1.00 |  |
| Agent State [Interactive] * Eye Contact | 1.22 | 1.13 – 1.32 |  |
| Group [ASD] * Eye Contact | 0.91 | 0.84 – 0.99 |  |
| **Random Effects** | | |  |
| σ^2^ | 3.29 | |  |
| τ_00_ _subj.ID_ | 0.38 | |  |
| ICC | 0.10 | |  |
| N _subj.ID_ | 45 | |  |
| Observations | 1636 | |  |
| Marginal R^2^ / Conditional R^2^ | 0.227 / 0.306 | |  |

**4.3 Effect of joint attention instances**

Analysis of the effect of the agents’ *interactivity*, diagnostic *group* and the number of joint attention instances on the participants probability to rate the agent interactive

Table S4.3.1: Likelihood ratio tests

| Model | Df | logLik | deviance | Chisq | Sig. |
| --- | --- | --- | --- | --- | --- |
| Interactivity * Group | 5 | -1006.55 | 2013.11 | - | - |
| Interactivity * Group + Joint Attention | 6 | -955.83 | 1911.66 | 101.45 | 0.000 |
| Interactivity * Group + Interactivity * Joint Attention | 7 | -917.56 | 1835.12 | 76.55 | 0.000 |
| Interactivity * Group + Interactivity * Joint Attention + Group * Joint Attention | 8 | -913.60 | 1827.21 | 7.91 | 0.005 |
| Interactivity * Group * Joint Attention | 9 | -909.14 | 1818.29 | 8.92 | 0.003 |

Table S4.3.2: Parameters of the model “Interactivity * Group * Joint Attention”

|  | |  | |  |
| --- | --- | --- | --- | --- |
| *Predictors* | | *Odds Ratios* | *CI* |  |
| (Intercept) | | 0.37 | 0.26 – 0.53 |  |
| Agent State [Interactive] | | 2.07 | 1.40 – 3.06 |  |
| Group [ASD] | | 1.09 | 0.64 – 1.85 |  |
| Joint Attention | | 1.18 | 1.07 – 1.30 |  |
| Agent State [Interactive] * Group [ASD] | | 0.95 | 0.54 – 1.65 |  |
| Agent State [Interactive] * Joint Attention | | 2.60 | 1.99 – 3.40 |  |
| Group [ASD] * Joint Attention | | 0.91 | 0.77 – 1.07 |  |
| Agent State [Interactive] * Group [ASD] * Joint Attention | | 0.61 | 0.43 – 0.85 |  |
| **Random Effects** | | | |  |
| σ^2^ | 3.29 | | |  |
| τ_00_ _subj.ID_ | 0.40 | | |  |
| ICC | 0.11 | | |  |
| N _subj.ID_ | 45 | | |  |
| Observations | 1636 | | |  |
| Marginal R^2^ / Conditional R^2^ | 0.435 / 0.496 | | |  |
